# 4-[4-(Diethyl­amino)­phen­yl]-*N*-methyl-3-nitro-4*H*-chromen-2-amine

**DOI:** 10.1107/S1600536811017338

**Published:** 2011-05-14

**Authors:** J. Muthukumaran, A. Parthiban, P. Manivel, H. Surya Prakash Rao, R. Krishna

**Affiliations:** a Centre for Bioinformatics, School of Life Sciences, Pondicherry University, Puducherry 605 014, India; b Department of Chemistry, Pondicherry University, Puducherry 605 014, India

## Abstract

In the title compound, C_20_H_23_N_3_O_3_, the dihydro­pyran ring adopts half-chair conformation. The chromene system makes a dihedral angle of 87.35 (5)° with the adjacent benzene ring. An intra­molecular N—H⋯O hydrogen bond generates an *S*(6) motif, which stabilizes the mol­ecular conformation. In the crystal, weak inter­molecular C—H⋯O hydrogen bonds contribute to the stabilization of the packing.

## Related literature

For the biological importance of 4*H*-chromene derivatives, see: Cai (2007 ▶, 2008 ▶); Cai *et al.* (2006 ▶); Gabor (1988 ▶); Brooks (1998 ▶); Valenti *et al.* (1993 ▶); Hyana & Saimoto (1987 ▶); Afanti­tis *et al.* (2006 ▶); Tang *et al.* (2007 ▶). For the structures of 4*H*-chromene derivatives, see: Muthukumaran *et al.* (2011 ▶); Gayathri *et al.* (2006 ▶); Bhaskaran *et al.* (2006 ▶). For ring puckering analysis, see: Cremer & Pople (1975 ▶) and for hydrogen-bond motifs, see: Bernstein *et al.* (1995 ▶).
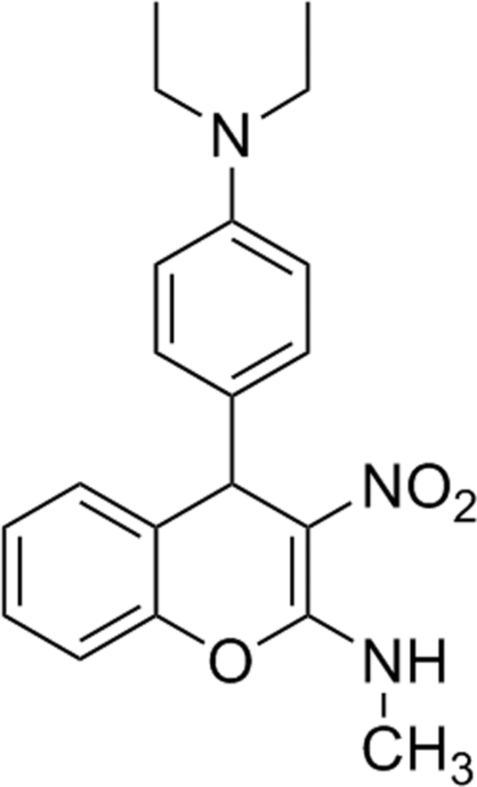


## Experimental

### 

#### Crystal data

C_20_H_23_N_3_O_3_
*M* *_r_* = 353.41
Triclinic, 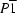

*a* = 8.9199 (11) Å
*b* = 10.4333 (12) Å
*c* = 11.6697 (8) Å
α = 65.100 (9)°
β = 82.388 (8)°
γ = 69.513 (11)°
*V* = 922.63 (19) Å^3^
*Z* = 2
Mo *K*α radiation
μ = 0.09 mm^−1^
*T* = 293 K
0.45 × 0.35 × 0.35 mm

#### Data collection

Oxford Diffraction Xcalibur Eos diffractometer
Absorption correction: multi-scan (*CrysAlis PRO*; Oxford Diffraction, 2009 ▶) *T*_min_ = 0.923, *T*_max_ = 1.000
17119 measured reflections
3242 independent reflections
2625 reflections with *I* > 2σ(*I*)
*R*_int_ = 0.036

#### Refinement

*R*[*F* ^2^ > 2σ(*F* ^2^)] = 0.056
*wR*(*F* ^2^) = 0.162
*S* = 1.05
3242 reflections
226 parameters
3 restraints
H-atom parameters constrained
Δρ_max_ = 0.52 e Å^−3^
Δρ_min_ = −0.44 e Å^−3^

### 

Data collection: *CrysAlis CCD* (Oxford Diffraction, 2009 ▶); cell refinement: *CrysAlis RED* (Oxford Diffraction, 2009 ▶); data reduction: *CrysAlis RED*; program(s) used to solve structure: *SHELXS97* (Sheldrick, 2008 ▶); program(s) used to refine structure: *SHELXL97* (Sheldrick, 2008 ▶); molecular graphics: *ORTEP-3 for Windows* (Farrugia, 1997 ▶) and *PLATON* (Spek, 2009 ▶); software used to prepare material for publication: *PLATON*.

## Supplementary Material

Crystal structure: contains datablocks I, global. DOI: 10.1107/S1600536811017338/sj5141sup1.cif

Structure factors: contains datablocks I. DOI: 10.1107/S1600536811017338/sj5141Isup2.hkl

Supplementary material file. DOI: 10.1107/S1600536811017338/sj5141Isup3.cml

Additional supplementary materials:  crystallographic information; 3D view; checkCIF report

## Figures and Tables

**Table 1:** Hydrogen-bond geometry (Å, °)

*D*—H⋯*A*	*D*—H	H⋯*A*	*D*⋯*A*	*D*—H⋯*A*
N1—H1⋯O2	0.86	1.96	2.596 (2)	129
C5—H5⋯O3^i^	0.93	2.52	3.325 (3)	144

Symmetry code: (i) 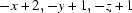.

**Fig. 1.**
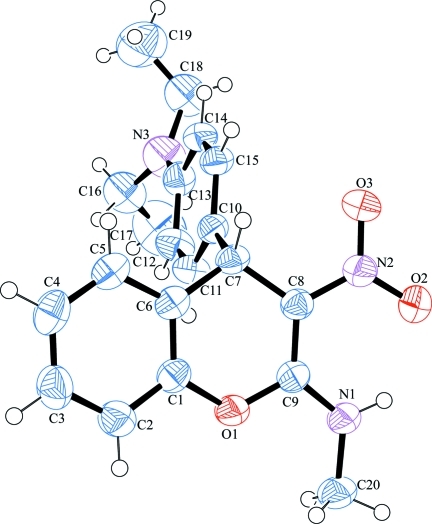
: The molecular structure of (I), showing displacement ellipsoids drawn at the 50% probability level.

**Fig. 2.**
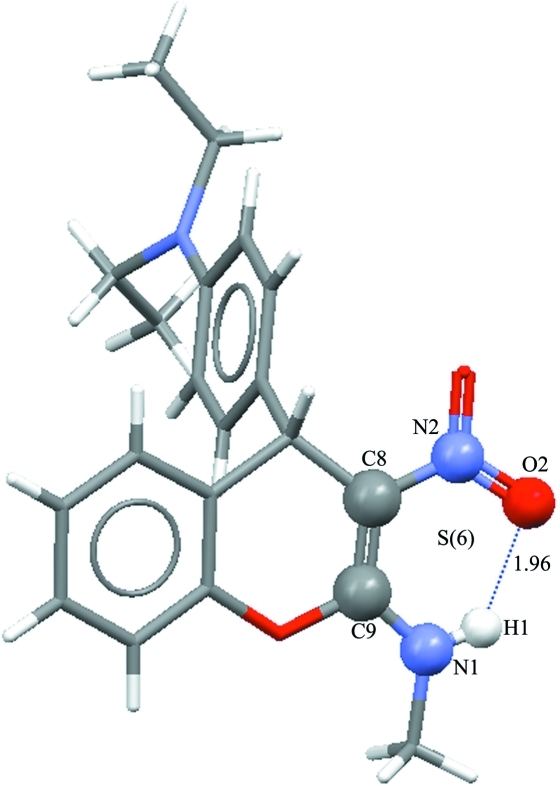
: A view of intramolecular motif S (6) formed by N—H···O interaction in Compound (I). The motif forming atoms are shown in ball and stick model and the Hydrogen bond are shown in blue dashed lines.

**Fig. 3.**
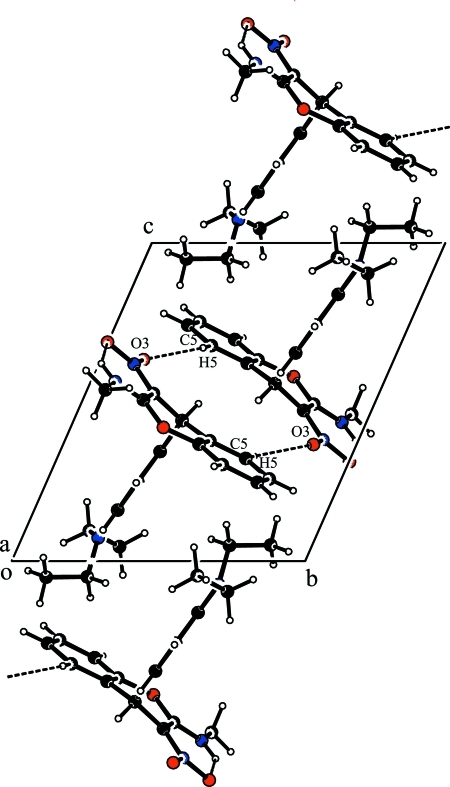
: The crystal packing of (I) showing intermolecular interactions as dashed lines.

## supplementary crystallographic information

### Comment

4*H*-chromenes and their derivatives possess various biological and pharmacological properties such as anti-viral, anti-fungal, anti-inflammatory, antidiabetic, cardionthonic, anti-anaphylactic and anti-cancer activity (Cai, 2008; Cai, 2007; Cai *et al.*, 2006; Gabor,1988; Brooks,1998; Valenti *et al.*, 1993; Hyana & Saimoto, 1987; Tang *et al.*, 2007). 4-aryl-4*H*-chromenes are a new series of apoptosis inducers, which exhibit potent anticancer activity (Afantitis *et al.*, 2006). Considering the importance of 4-aryl-4*H*-chromene derivatives, a single-crystal X-ray diffraction study on the title compound was carried out and analyzed.

Some 4*H*-chromene derivatives are already reported in the literature (Muthukumaran *et al.*, 2011; Gayathri *et al.*, 2006; Bhaskaran *et al.*, 2006). The molecular structure of the title compound is shown in Fig. 1. From the puckering analysis (Cremer & Pople, 1975), the fused dihydropyran ring (O1/C1/C6/C7/C8/C9) of 4*H*-chromene system is very similar to half chair (H form) conformation with puckering parameters of Q = 0.253 (2) Å, θ = 103.2 (5) ° and Φ = 7.0 (5) °. In the title compound, the 4*H*-chromene system makes a dihedral angle of 87.35 (5)° with the adjacent phenyl ring. The intramolecular N1—H1···O2 interaction generates a graph-set motif S (6) (Bernstein *et al.*, 1995) (Fig. 2) with a *D···A* bond distance of 2.596 (2) Å. The crystal packing (Fig. 3) is stabilized by weak intermolecular C—H···O interactions.

### Experimental

To a vigorously stirred solution of *N*-methyl-*N*-[3-nitro-4-(methylsulfanyl)-4*H-*2-chromenyl]amine (0.5 g, 2 mmol) in ethanol (15 ml), *N*, *N*-diethylaminobenzene (0.33 g, 2.2 mmol) was added and the resulting solution was refluxed for 12 h by which time the reaction was complete (TLC; hexane: EtOAc, 6:4). The reaction mixture was cooled to room temperature and kept aside for 3 h. The solid, which separated was filtered to obtain 0.59 g of *N*2-methyl-4-[4-(diethylamino)phenyl]-3-nitro-4*H*-2-chromenamine in 92% yield as colorless solid; mp 201 °C. *R*_f_ 0.4 (hexane: EtOAc, 6:4). A sample suitable for single crystal X-ray analysis was obtained by recrystallization from a mixture of dichloromethane and hexane (3:1).

### Refinement

All hydrogen atoms were placed in calculated positions, with N—H=0.86 and C—H=0.93 and included in the final cycles of refinement using a riding model with *U*_iso_(H) = 1.2 *U*_eq_(C).

### Crystal data

**Table:**

C_20_H_23_N_3_O_3_	*Z* = 2
*M**_r_* = 353.41	*F*(000) = 376
Triclinic, *P*1	*D*_x_ = 1.272 Mg m^−^^3^
Hall symbol: -P 1	Mo *K*α radiation, λ = 0.71073 Å
*a* = 8.9199 (11) Å	Cell parameters from 8151 reflections
*b* = 10.4333 (12) Å	θ = 2.7–29.3°
*c* = 11.6697 (8) Å	µ = 0.09 mm^−^^1^
α = 65.100 (9)°	*T* = 293 K
β = 82.388 (8)°	Block, yellow
γ = 69.513 (11)°	0.45 × 0.35 × 0.35 mm
*V* = 922.63 (19) Å^3^	

### Data collection

**Table:**

Oxford Diffraction Xcalibur Eos diffractometer	3242 independent reflections
Radiation source: fine-focus sealed tube	2625 reflections with *I* > 2σ(*I*)
graphite	*R*_int_ = 0.036
Detector resolution: 15.9821 pixels mm^-1^	θ_max_ = 25.0°, θ_min_ = 2.7°
ω scans	*h* = −10→10
Absorption correction: multi-scan (*CrysAlis PRO*; Oxford Diffraction, 2009)	*k* = −12→12
*T*_min_ = 0.923, *T*_max_ = 1.000	*l* = −13→13
17119 measured reflections	

### Refinement

**Table:**

Refinement on *F*^2^	Primary atom site location: structure-invariant direct methods
Least-squares matrix: full	Secondary atom site location: difference Fourier map
*R*[*F*^2^ > 2σ(*F*^2^)] = 0.056	Hydrogen site location: inferred from neighbouring sites
*wR*(*F*^2^) = 0.162	H-atom parameters constrained
*S* = 1.05	*w* = 1/[σ^2^(*F*_o_^2^) + (0.0737*P*)^2^ + 0.5254*P*] where *P* = (*F*_o_^2^ + 2*F*_c_^2^)/3
3242 reflections	(Δ/σ)_max_ < 0.001
226 parameters	Δρ_max_ = 0.52 e Å^−^^3^
3 restraints	Δρ_min_ = −0.44 e Å^−^^3^

### Special details

**Table:**

Geometry. All e.s.d.'s (except the e.s.d. in the dihedral angle between two l.s. planes) are estimated using the full covariance matrix. The cell e.s.d.'s are taken into account individually in the estimation of e.s.d.'s in distances, angles and torsion angles; correlations between e.s.d.'s in cell parameters are only used when they are defined by crystal symmetry. An approximate (isotropic) treatment of cell e.s.d.'s is used for estimating e.s.d.'s involving l.s. planes.
Refinement. Refinement of *F*^2^ against ALL reflections. The weighted *R*-factor *wR* and goodness of fit *S* are based on *F*^2^, conventional *R*-factors *R* are based on *F*, with *F* set to zero for negative *F*^2^. The threshold expression of *F*^2^ > σ(*F*^2^) is used only for calculating *R*-factors(gt) *etc*. and is not relevant to the choice of reflections for refinement. *R*-factors based on *F*^2^ are statistically about twice as large as those based on *F*, and *R*- factors based on ALL data will be even larger.

### Fractional atomic coordinates and isotropic or equivalent isotropic displacement parameters (Å^2^)

**Table:**

	*x*	*y*	*z*	*U*_iso_*/*U*_eq_	
O1	0.42869 (17)	0.68371 (16)	0.57975 (15)	0.0483 (4)	
N1	0.3634 (2)	0.9167 (2)	0.43607 (18)	0.0466 (5)	
H1	0.3882	0.9910	0.3790	0.056*	
N2	0.6967 (2)	0.8783 (2)	0.38082 (17)	0.0474 (5)	
C8	0.6437 (2)	0.7689 (2)	0.47334 (19)	0.0395 (5)	
C7	0.7689 (2)	0.6305 (2)	0.55805 (19)	0.0391 (5)	
H7	0.8571	0.6001	0.5053	0.047*	
O3	0.84395 (19)	0.8532 (2)	0.36670 (17)	0.0620 (5)	
C9	0.4810 (2)	0.7932 (2)	0.4932 (2)	0.0397 (5)	
O2	0.5993 (2)	1.00198 (19)	0.31188 (17)	0.0658 (5)	
C6	0.6982 (2)	0.5058 (2)	0.6218 (2)	0.0399 (5)	
C10	0.8361 (2)	0.6586 (2)	0.65559 (19)	0.0385 (5)	
C15	0.9923 (2)	0.6551 (2)	0.6541 (2)	0.0425 (5)	
H15	1.0599	0.6340	0.5919	0.051*	
C1	0.5350 (3)	0.5370 (2)	0.6324 (2)	0.0424 (5)	
C13	0.9558 (3)	0.7122 (3)	0.8384 (2)	0.0491 (6)	
C5	0.7938 (3)	0.3569 (3)	0.6773 (2)	0.0484 (6)	
H5	0.9044	0.3321	0.6705	0.058*	
C11	0.7400 (3)	0.6897 (3)	0.7504 (2)	0.0482 (5)	
H11	0.6339	0.6932	0.7536	0.058*	
C14	1.0514 (3)	0.6821 (3)	0.7422 (2)	0.0478 (5)	
H14	1.1570	0.6802	0.7373	0.057*	
C12	0.7968 (3)	0.7153 (3)	0.8397 (2)	0.0544 (6)	
H12	0.7288	0.7351	0.9022	0.065*	
C20	0.1945 (3)	0.9377 (3)	0.4617 (3)	0.0563 (6)	
H20A	0.1681	0.8589	0.4560	0.084*	
H20B	0.1316	1.0323	0.4009	0.084*	
H20C	0.1724	0.9357	0.5451	0.084*	
N3	1.0125 (3)	0.7401 (3)	0.9267 (2)	0.0778 (5)	
C2	0.4655 (3)	0.4275 (3)	0.6966 (2)	0.0527 (6)	
H2	0.3548	0.4520	0.7021	0.063*	
C3	0.5633 (3)	0.2813 (3)	0.7523 (3)	0.0602 (7)	
H3	0.5187	0.2061	0.7971	0.072*	
C4	0.7278 (3)	0.2458 (3)	0.7418 (2)	0.0584 (6)	
H4	0.7935	0.1467	0.7784	0.070*	
C18	1.1609 (4)	0.7810 (4)	0.9042 (3)	0.0778 (5)	
H18A	1.1720	0.8322	0.8140	0.093*	
H18B	1.1496	0.8506	0.9421	0.093*	
C16	0.9352 (4)	0.7188 (4)	1.0492 (3)	0.0778 (5)	
H16A	0.8790	0.6478	1.0685	0.093*	
H16B	1.0165	0.6772	1.1144	0.093*	
C17	0.8226 (6)	0.8584 (5)	1.0503 (5)	0.1329 (17)	
H17A	0.8774	0.9296	1.0298	0.199*	
H17B	0.7774	0.8405	1.1327	0.199*	
H17C	0.7387	0.8971	0.9890	0.199*	
C19	1.3069 (5)	0.6543 (5)	0.9545 (4)	0.1141 (14)	
H19A	1.2959	0.6005	1.0433	0.171*	
H19B	1.3960	0.6901	0.9411	0.171*	
H19C	1.3248	0.5891	0.9120	0.171*	

### Atomic displacement parameters (Å^2^)

**Table:**

	*U*^11^	*U*^22^	*U*^33^	*U*^12^	*U*^13^	*U*^23^
O1	0.0341 (8)	0.0406 (8)	0.0607 (10)	−0.0087 (6)	0.0025 (7)	−0.0150 (7)
N1	0.0364 (9)	0.0393 (10)	0.0554 (11)	−0.0078 (8)	−0.0005 (8)	−0.0145 (9)
N2	0.0400 (10)	0.0525 (11)	0.0445 (11)	−0.0133 (9)	0.0030 (8)	−0.0168 (9)
C8	0.0367 (11)	0.0437 (11)	0.0381 (11)	−0.0115 (9)	0.0013 (9)	−0.0179 (9)
C7	0.0316 (10)	0.0437 (11)	0.0406 (11)	−0.0070 (8)	0.0014 (8)	−0.0202 (9)
O3	0.0406 (9)	0.0702 (11)	0.0623 (11)	−0.0191 (8)	0.0105 (8)	−0.0170 (9)
C9	0.0386 (11)	0.0401 (11)	0.0411 (11)	−0.0103 (9)	−0.0002 (9)	−0.0187 (9)
O2	0.0520 (10)	0.0530 (10)	0.0628 (11)	−0.0111 (8)	−0.0009 (8)	−0.0005 (9)
C6	0.0407 (11)	0.0436 (12)	0.0386 (11)	−0.0100 (9)	−0.0032 (9)	−0.0214 (9)
C10	0.0361 (10)	0.0386 (11)	0.0383 (11)	−0.0094 (8)	−0.0008 (8)	−0.0150 (9)
C15	0.0344 (11)	0.0470 (12)	0.0442 (12)	−0.0094 (9)	0.0013 (9)	−0.0199 (10)
C1	0.0418 (11)	0.0393 (11)	0.0451 (12)	−0.0090 (9)	−0.0023 (9)	−0.0186 (10)
C13	0.0539 (13)	0.0516 (13)	0.0449 (13)	−0.0201 (11)	−0.0051 (10)	−0.0184 (11)
C5	0.0455 (12)	0.0486 (13)	0.0494 (13)	−0.0057 (10)	−0.0061 (10)	−0.0241 (11)
C11	0.0381 (11)	0.0626 (14)	0.0517 (13)	−0.0198 (10)	0.0060 (10)	−0.0292 (11)
C14	0.0371 (11)	0.0543 (13)	0.0515 (13)	−0.0157 (10)	−0.0025 (10)	−0.0192 (11)
C12	0.0543 (14)	0.0726 (16)	0.0496 (14)	−0.0261 (12)	0.0123 (11)	−0.0359 (13)
C20	0.0363 (12)	0.0499 (13)	0.0732 (17)	−0.0054 (10)	0.0002 (11)	−0.0229 (12)
N3	0.0905 (12)	0.1014 (13)	0.0670 (10)	−0.0482 (10)	−0.0023 (9)	−0.0428 (10)
C2	0.0494 (13)	0.0516 (14)	0.0595 (15)	−0.0204 (11)	0.0012 (11)	−0.0216 (12)
C3	0.0729 (17)	0.0459 (14)	0.0617 (16)	−0.0251 (12)	−0.0033 (13)	−0.0158 (12)
C4	0.0681 (17)	0.0405 (13)	0.0597 (15)	−0.0073 (11)	−0.0104 (12)	−0.0191 (11)
C18	0.0905 (12)	0.1014 (13)	0.0670 (10)	−0.0482 (10)	−0.0023 (9)	−0.0428 (10)
C16	0.0905 (12)	0.1014 (13)	0.0670 (10)	−0.0482 (10)	−0.0023 (9)	−0.0428 (10)
C17	0.172 (5)	0.129 (4)	0.145 (4)	−0.071 (3)	0.028 (3)	−0.088 (3)
C19	0.094 (3)	0.151 (4)	0.098 (3)	−0.039 (3)	−0.022 (2)	−0.044 (3)

### Geometric parameters (Å, °)

**Table:**

O1—C9	1.349 (3)	C11—C12	1.373 (3)
O1—C1	1.402 (2)	C11—H11	0.9300
N1—C9	1.312 (3)	C14—H14	0.9300
N1—C20	1.453 (3)	C12—H12	0.9300
N1—H1	0.8600	C20—H20A	0.9600
N2—O3	1.249 (2)	C20—H20B	0.9600
N2—O2	1.262 (2)	C20—H20C	0.9600
N2—C8	1.377 (3)	N3—C16	1.465 (4)
C8—C9	1.388 (3)	N3—C18	1.487 (4)
C8—C7	1.507 (3)	C2—C3	1.376 (3)
C7—C6	1.510 (3)	C2—H2	0.9300
C7—C10	1.525 (3)	C3—C4	1.384 (4)
C7—H7	0.9800	C3—H3	0.9300
O3—N2	1.249 (2)	C4—H4	0.9300
O2—N2	1.262 (2)	C18—C19	1.460 (5)
C6—C1	1.377 (3)	C18—H18A	0.9700
C6—C5	1.390 (3)	C18—H18B	0.9700
C10—C15	1.380 (3)	C16—C17	1.455 (5)
C10—C11	1.385 (3)	C16—H16A	0.9700
C15—C14	1.380 (3)	C16—H16B	0.9700
C15—H15	0.9300	C17—H17A	0.9600
C1—C2	1.380 (3)	C17—H17B	0.9600
C13—N3	1.378 (3)	C17—H17C	0.9600
C13—C14	1.393 (3)	C19—H19A	0.9600
C13—C12	1.406 (3)	C19—H19B	0.9600
C5—C4	1.372 (4)	C19—H19C	0.9600
C5—H5	0.9300		
			
C9—O1—C1	119.79 (16)	C11—C12—H12	119.4
C9—N1—C20	125.1 (2)	C13—C12—H12	119.4
C9—N1—H1	117.5	N1—C20—H20A	109.5
C20—N1—H1	117.5	N1—C20—H20B	109.5
O3—N2—O2	120.32 (18)	H20A—C20—H20B	109.5
O3—N2—O2	120.32 (18)	N1—C20—H20C	109.5
O3—N2—C8	118.58 (18)	H20A—C20—H20C	109.5
O2—N2—C8	121.10 (18)	H20B—C20—H20C	109.5
N2—C8—C9	120.35 (19)	C13—N3—C16	120.7 (2)
N2—C8—C7	117.15 (17)	C13—N3—C18	120.8 (2)
C9—C8—C7	122.25 (19)	C16—N3—C18	118.3 (2)
C6—C7—C8	109.37 (17)	C3—C2—C1	118.6 (2)
C6—C7—C10	110.83 (17)	C3—C2—H2	120.7
C8—C7—C10	111.95 (17)	C1—C2—H2	120.7
C6—C7—H7	108.2	C2—C3—C4	120.2 (2)
C10—C7—H7	108.2	C2—C3—H3	119.9
N1—C9—O1	112.50 (18)	C4—C3—H3	119.9
N1—C9—C8	127.1 (2)	C5—C4—C3	119.9 (2)
O1—C9—C8	120.41 (18)	C5—C4—H4	120.0
C1—C6—C5	117.4 (2)	C3—C4—H4	120.0
C1—C6—C7	120.57 (18)	C19—C18—N3	114.4 (3)
C5—C6—C7	121.92 (19)	C19—C18—H18A	108.7
C15—C10—C11	117.06 (19)	N3—C18—H18A	108.7
C15—C10—C7	122.49 (18)	C19—C18—H18B	108.7
C11—C10—C7	120.45 (18)	N3—C18—H18B	108.7
C14—C15—C10	122.0 (2)	H18A—C18—H18B	107.6
C14—C15—H15	119.0	C17—C16—N3	112.0 (3)
C10—C15—H15	119.0	C17—C16—H16A	109.2
C6—C1—C2	122.6 (2)	N3—C16—H16A	109.2
C6—C1—O1	121.69 (19)	C17—C16—H16B	109.2
C2—C1—O1	115.70 (19)	N3—C16—H16B	109.2
N3—C13—C14	122.1 (2)	H16A—C16—H16B	107.9
N3—C13—C12	121.4 (2)	C16—C17—H17A	109.5
C14—C13—C12	116.5 (2)	C16—C17—H17B	109.5
C4—C5—C6	121.2 (2)	H17A—C17—H17B	109.5
C4—C5—H5	119.4	C16—C17—H17C	109.5
C6—C5—H5	119.4	H17A—C17—H17C	109.5
C12—C11—C10	121.9 (2)	H17B—C17—H17C	109.5
C12—C11—H11	119.0	C18—C19—H19A	109.5
C10—C11—H11	119.0	C18—C19—H19B	109.5
C15—C14—C13	121.3 (2)	H19A—C19—H19B	109.5
C15—C14—H14	119.3	C18—C19—H19C	109.5
C13—C14—H14	119.3	H19A—C19—H19C	109.5
C11—C12—C13	121.2 (2)	H19B—C19—H19C	109.5
			
O3—N2—C8—N2	0(17)	C8—C7—C6—C5	162.23 (19)
O2—N2—C8—N2	0(100)	C10—C7—C6—C5	−73.9 (2)
O2—N2—C8—N2	0(100)	C6—C7—C10—C15	124.8 (2)
N2—N2—C8—C9	0.00 (11)	C8—C7—C10—C15	−112.8 (2)
O3—N2—C8—C9	179.15 (19)	C6—C7—C10—C11	−55.4 (3)
O2—N2—C8—C9	−0.4 (3)	C8—C7—C10—C11	67.0 (3)
O2—N2—C8—C9	−0.4 (3)	C11—C10—C15—C14	−0.4 (3)
N2—N2—C8—C7	0.00 (19)	C7—C10—C15—C14	179.4 (2)
O3—N2—C8—C7	4.7 (3)	C5—C6—C1—C2	1.0 (3)
O2—N2—C8—C7	−174.88 (19)	C7—C6—C1—C2	−175.9 (2)
O2—N2—C8—C7	−174.88 (19)	C5—C6—C1—O1	179.92 (18)
N2—C8—C7—C6	−161.30 (17)	C7—C6—C1—O1	3.0 (3)
N2—C8—C7—C6	−161.30 (17)	C9—O1—C1—C6	15.4 (3)
C9—C8—C7—C6	24.4 (3)	C9—O1—C1—C2	−165.57 (19)
N2—C8—C7—C10	75.5 (2)	C1—C6—C5—C4	−1.0 (3)
N2—C8—C7—C10	75.5 (2)	C7—C6—C5—C4	175.8 (2)
C9—C8—C7—C10	−98.9 (2)	C15—C10—C11—C12	−0.3 (3)
O2—N2—O3—N2	0(39)	C7—C10—C11—C12	179.9 (2)
O2—N2—O3—N2	0(39)	C10—C15—C14—C13	1.0 (3)
C8—N2—O3—N2	0(100)	N3—C13—C14—C15	−179.6 (2)
C20—N1—C9—O1	0.6 (3)	C12—C13—C14—C15	−0.8 (3)
C20—N1—C9—C8	−178.9 (2)	C10—C11—C12—C13	0.4 (4)
C1—O1—C9—N1	168.16 (18)	N3—C13—C12—C11	179.0 (2)
C1—O1—C9—C8	−12.3 (3)	C14—C13—C12—C11	0.1 (4)
N2—C8—C9—N1	−3.7 (3)	C14—C13—N3—C16	−158.1 (3)
N2—C8—C9—N1	−3.7 (3)	C12—C13—N3—C16	23.1 (4)
C7—C8—C9—N1	170.5 (2)	C14—C13—N3—C18	17.1 (4)
N2—C8—C9—O1	176.92 (18)	C12—C13—N3—C18	−161.7 (3)
N2—C8—C9—O1	176.92 (18)	C6—C1—C2—C3	0.0 (4)
C7—C8—C9—O1	−8.9 (3)	O1—C1—C2—C3	−179.0 (2)
N2—N2—O2—O2	0.0	C1—C2—C3—C4	−1.0 (4)
O3—N2—O2—O2	0.0 (2)	C6—C5—C4—C3	0.1 (4)
C8—N2—O2—O2	0.00 (11)	C2—C3—C4—C5	1.0 (4)
O3—N2—O2—N2	0(10)	C13—N3—C18—C19	−92.4 (4)
O2—N2—O2—N2	0(100)	C16—N3—C18—C19	82.9 (4)
C8—N2—O2—N2	0(100)	C13—N3—C16—C17	−97.1 (4)
C8—C7—C6—C1	−21.0 (3)	C18—N3—C16—C17	87.6 (4)
C10—C7—C6—C1	102.9 (2)		

### Hydrogen-bond geometry (Å, °)

**Table:**

*D*—H···*A*	*D*—H	H···*A*	*D*···*A*	*D*—H···*A*
N1—H1···O2	0.86	1.96	2.596 (2)	129
C5—H5···O3^i^	0.93	2.52	3.325 (3)	144

Symmetry codes:  (i) −*x*+2, −*y*+1, −*z*+1.
